# Efficacy and Prognostic Factors of Androgen Deprivation Therapy Combined with Radiation Therapy for Prostate Cancer

**DOI:** 10.1155/2021/2237069

**Published:** 2021-11-09

**Authors:** Siping Zeng, Gangyun Guan, Qiuwei Qin, Huadong Xie, Yongyan Meng, Qiyue Zhao

**Affiliations:** ^1^Department of Urology, Liuzhou Workers' Hospital, Liuzhou 545007, Guangxi Zhuang Autonomous Region, China; ^2^Department of Obstetrics and Gynecology, Xiangzhou People's Hospital, Laibin 545800, Guangxi Zhuang Autonomous Region, China

## Abstract

**Objective:**

To analyze the efficacy of androgen deprivation therapy (ADT) combined with radiation therapy (also known as radiotherapy) for prostate cancer.

**Methods:**

The clinical data of 94 prostate cancer patients treated in the Oncology Department of Xiangzhou People's Hospital from January 2017 to January 2018 were retrospectively analyzed, and the patients were divided into the combined group and the reference group according to their admission order, with 47 cases each. The patients in the reference group only received the radiotherapy, and on this basis, those in the combined group accepted ADT, so as to evaluate the efficacy of different treatment methods by comparing the patients' serum total prostate-specific antigen (T-PSA), vascular endothelial growth factor (VEGF), and other indicators and analyze the relevant factors affecting patients' prognosis by Cox single-factor and multi-factor regression models.

**Results:**

Compared with the reference group after treatment, the patients in the combined group obtained significantly lower T-PSA and VEGF levels (*P* < 0.001), significantly higher objective remission rate and disease control rate (*P* < 0.05), and remarkably longer modified progression-free survival (mPFS) and overall survival (OS) (*P* < 0.001), and after the multi-factor research, it was found that the Gleason score of 8–10, positive lymphatic metastasis, and single radiotherapy were the factors affecting the clinical prognosis of prostate cancer.

**Conclusion:**

Combining ADT with radiotherapy ensures a better survival benefit for prostate cancer patients and has a fairly well efficacy. Further study will be conducive to establishing a better solution for such patients.

## 1. Introduction

Authoritative survey data show [1] that the incidence of prostate cancer reaches the 5^th^ place among malignant tumors, becoming one of the important diseases endangering the male population. The tumor has an insidious onset and slow growth and will trigger dysuria, interruption of urinary stream, hematuria, and other symptoms once it causes swelling of the prostate, resulting in high deterioration, difficulty in treatment, and poor clinical prognosis and seriously affecting the physical and psychological functions of patients. It is generally believed that radical surgery is a more thorough treatment for prostate cancer but because the initial symptoms are not obvious, most diagnosed patients are often inoperable because of the late pathological stage and older age [2–4]. With the continuous improvement of radiotherapy technology, precise and adaptable radiotherapy treatments are widely recommended in the clinic to benefit patients [5]. However, radiotherapy can damage the body's immune system and then lead to decreased immune function, and most patients also experience nausea and vomiting and other gastrointestinal reactions, affecting the treatment effect. Previous studies have found [6] that this neoplastic disease is an androgen-dependent malignancy and that cancer cells undergo apoptosis in the absence of androgen stimulation, and therefore the inhibition of disease progression can be achieved by reducing androgen levels. Some foreign scholars [7] found that surgical castration and androgen deprivation can delay the progression of metastatic prostate cancer to some extent, thereby pioneering the hormonal therapy. Currently, there are many reports on androgen castration therapy and radiotherapy for prostate cancer, but few focus on the efficacy and prognostic factors of the combination of the two for prostate cancer patients. Based on this, a control study was carried out herein to explore the efficacy and prognostic factors of the combined therapy for prostate cancer patients, with the results reported as follows.

## 2. Case Data and Methods

### 2.1. General Information

The clinical data of 94 prostate cancer patients treated in the Oncology Department of Xiangzhou People's Hospital from January 2017 to January 2018 were retrospectively analyzed, and the patients were divided into the combined group and the reference group according to their admission order, with 47 cases each. This study was approved by the ethics committee of Xiangzhou People's Hospital.

### 2.2. Inclusion and Exclusion Criteria

#### 2.2.1. Inclusion Criteria

① The enrolled patients met the diagnosis criteria for prostate cancer in the 2018 version of Specification for Diagnosis and Treatment of Prostate Cancer [8], presented the clinical manifestations such as urgent micturition, frequent micturition, dysuria, and urinary incontinence, and were diagnosed after pathological examination; ② the patients' estimated survival was more than one year and their clinical data were complete; ③ the patients obtained over 70 points in the KPS score and not greater than 2 points in the U.S. Eastern Cooperative Oncology Group (ECOG) score [9], did not have radiotherapy contraindications, and could accept the entire treatment; ④ the patients accepted relevant immunotherapy and hormonal therapy; and ⑤ the patients or their statutory guardians understood the study process and signed the informed consent.

#### 2.2.2. Exclusion Criteria

① The Gleason score was less than 7 points; ② the patients suffered from other malignant tumors or immune dysfunction; ③ the patients presented severe cardiovascular and cerebrovascular diseases, viral hepatitis, or other chronic hepatic diseases; and ④ the patients presented abnormal mental state and could not go through the study.

### 2.3. Methods

The patients in the reference group only received the image-guided radiation therapy (IGRT) with the following steps. The CT scanning simulation was conducted after fixing the phantom, the patient's position, ISO-center, and reference points on skin were determined under the CT scan simulator, and the exposure range included the whole pelvis, local prostate, and pelvic lymph nodes [10]. The median radiation dose was 74 Gy, and four fields were radiated daily. For the whole pelvis, it was 2 Gy each time, 5 times a week for 5 weeks (total 25 times). Then, the fields were shrunk to the local prostate with a supplementary dose of 24 Gy, and the dose of planning target volume (PTV) was 1.8–2.0 Gy each time and 5 times a week for 2–3 weeks. The treatment was discontinued in case of grade III or above adverse reactions. During treatment, drugs to relieve vomiting, protective agents for gastric mucosa, and granulocyte colony stimulating factor were applied according to the patients' symptoms. After radiation therapy, the patients received regular follow-up checks [11, 12].

On the basis of radiation therapy, the patients in the combined group received ADT with the following steps. 3.6 mg of goserelin acetate sustained-release implant (manufactured: AstraZeneca UK Limited; registration no. H20100314; specification: 3.6 mg × one dose) was administered subcutaneously in the anterior abdominal wall every 28 days. Meanwhile, for anti-androgen hormones, 150 mg of bicalutamide tablets (manufactured: AstraZeneca UK Limited; NMPA approval no. J20150050; specification: 50 mg *∗* 28 s) was taken orally 30 min after meal once a day for 2–4 weeks. The patients' liver function and blood routine parameters were checked regularly. The treatment should be stopped in case of any liver dysfunction (jaundice, cholestasis, and elevation of aminotransferase).

### 2.4. Evaluation Indexes

#### 2.4.1. Serum Indicator Detection

Fasting elbow venous blood (5 ml) was collected from the patients in both groups after treatment and centrifuged with the centrifugal machine (manufactured: Jinan OLABO Technology Co., Ltd.; model: TD-4X) under 3,000 r/min for 15 min to separate serum for test, T-PSA level values of patients before and after treatment were determined by electrochemiluminescence assay, and VEGF level values were determined by immunosorbent assay, with the kits provided by Beijing Aviva Systems Biology Co., Ltd. The operation procedures were performed according to the kit instructions.

#### 2.4.2. Efficacy Determination

The clinical efficacy after treatment of patients in the two groups was evaluated by referring to the Response Evaluation Criteria in Solid Tumors (RECIST) 1.1 [13] recommended by the World Health Organization (WHO), and the number of cases with complete response (CR), partial response (PR), stable disease (SD), and progressive disease (PD) was counted. The T-PSA level values in patients after treatment were regarded as the evaluation criterion for efficacy, i.e., referring to the percentage of the PSA difference after treatment divided by the T-PSA value before treatment, the reduction rate ≥75% indicated CR, 75% < reduction rate ≤50% indicated PR, 49% < reduction rate ≤30% indicated SD, and reduction rate <29% indicated PD; the disease control rate (DCR) = (CR + PR + SD) cases/total number × 100%, and the objective remission rate (ORR) = (CR + PR) cases/total number × 100%.

#### 2.4.3. Follow-Up Observation

The researchers obtained clinical data and pathological characteristics by reviewing the patients' medical records in detail and by telephone follow-up with the patients or their legal guardians, and all patients were regularly reviewed for CT scans and tumor markers to record their overall survival (OS) and disease progression-free survival (PFS).

### 2.5. Statistical Methods

In this study, the data were analyzed by the statistic software SPSS 26.0, the measurement data were expressed by (mean ± SD) and examined by *t*-test, the enumeration data were expressed by *n*(%) and examined by *X*^2^ test, the factors affecting patients' prognosis were analyzed by single-factor and multi-factor Cox proportional-hazards regression models, and differences were considered statistically significant at *P* < 0.05.

## 3. Results

### 3.1. Between-Group Comparison of Baseline Data

No significant differences in the mean age, BMI values, prostate volume, TNM pathological stage, and other general information between the two groups were observed (*P* > 0.05) (see Table 1).

### 3.2. Between-Group Comparison of Serum T-PSA and VEGF Level Values after Treatment

**Figure 1:**
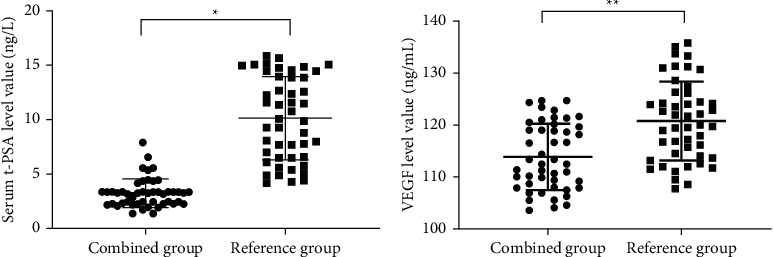
Between-group comparison of serum T-PSA and VEGF level values after treatment (mean ± SD). (a) The between-group comparison of serum T-PSA level values after treatment. The horizontal axis denoted the combined group and the reference group, and the vertical axis denoted the value (ng/mL). After treatment, the mean serum T-PSA level values of the combined group and the reference group were (3.15 ± 1.32) and (10.07 ± 3.84), respectively, and *∗* indicated that the mean serum T-PSA level values after treatment between the two groups were significantly different (*t* = 11.683, *P* < 0.001). (b) The between-group comparison of serum VEGF level values after treatment. The horizontal axis denoted the combined group and the reference group, and the vertical axis denoted the value (ng/mL). After treatment, the mean serum VEGF level values of the combined group and the reference group were (113.90 ± 6.41) and (120.84 ± 7.61), respectively, and ^∗∗^ indicated that the mean serum VEGF level values after treatment between the two groups were significantly different (*t* = 4.782, *P* < 0.001).

After treatment, the serum T-PSA and VEGF levels were significantly lower in the combined group than in the reference group (*P* < 0.001) (see Figure 1).

### 3.3. Between-Group Comparison of Treatment Effect

Compared with the reference group after treatment, the combined group obtained remarkably higher ORR and DCR (*P* < 0.05) (see Table 2).

### 3.4. Between-Group Comparison of mPFS and OS

**Figure 2:**
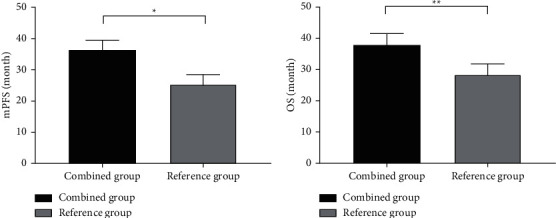
Between-group comparison of mPFS and OS (mean ± SD). (a) The between-group comparison of mPFS. The horizontal axis denoted the combined group and the reference group, and the vertical axis denoted mPFS (month). The mPFS of the combined group and the reference group was (36.23 ± 3.27) and (25.36 ± 3.53), respectively, and *∗* indicated significant difference in mPFS between the two groups (*t* = 15.487, *P* < 0.001). (b) The between-group comparison of OS. The horizontal axis denoted the combined group and the reference group, and the vertical axis denoted OS (month). The OS of the combined group and the reference group was (38.17 ± 3.43) and (28.44 ± 3.46), respectively, and ^∗∗^ indicated significant difference in OS between the two groups (*t* = 13.692, *P* < 0.001).

The mPFS and OS were significantly longer in the combined group than in the reference group (*P* < 0.001) (see Figure 2).

### 3.5. Single-Factor and Multi-Factor Retrospective Analyses on Clinical Prognosis of Patients

It was found that single radiation therapy, Gleason score of 8–10, and positive lymphatic metastasis were the factors affecting patients' clinical prognosis (see Tables 3 and 4).

## 4. Discussion

Prostate cancer refers to the epithelial malignancy in the prostate [14, 15], which often occurs in people over 55 years old and is an important disease that endangers men's life and health. With the increasing worldwide aging population in recent years, its incidence is rising year by year, seriously affecting patients' physical health [16, 17]. Surgery is currently the main treatment for prostate cancer because the condition can be effectively controlled by tumor resection, but some patients are inoperable because of the late pathological stage, older age, and more comorbidities when diagnosed [18]. Radiotherapy has the advantages of significant efficacy and wide indications, further increasing the radiation dose to the cancer target area and reducing radiation damage to the surrounding tissues, which has been demonstrated in elderly patients with advanced prostate cancer [19]. But radiotherapy will adversely affect normal cells in the body while killing cancer cells and then cause immune function damage and affect rehabilitation. In recent years, studies have revealed a strong association between androgen in the human body and a number of prostate diseases. Based on this theory, the androgen castration, a unique treatment that targets the prostate, was developed [20], which completely blocks androgen in patients by drug action and achieves the goal of controlling or reducing the proliferation and spread of tumor cells. As a non-steroid drug, the bicalutamide tablets used in this study have better peripheral selectivity and can bind to the androgen receptor without effective gene expression, thus inhibiting the stimulation of androgens and causing the atrophy of prostate tumors. It is demonstrated [21] that more than 85% of low-risk prostate cancer patients who accepted the androgen castration obtain a 5-year postoperative survival rate; however, some scholars believe that although castration surgery can effectively block testis-derived androgen, the progression of prostate cancer can be promoted by the adrenal gland-secreted androgen. Therefore, most scholars agree that the androgen deprivation therapy with oral drugs is more effective, tolerable, and convenient [22, 23].

In this study, single radiotherapy and combining ADT with radiotherapy were implemented to 94 prostate cancer patients in the two groups, respectively, and the results showed that compared with the reference group, the clinical effect and serum indicators of the combined group were better. T-PSA is a glycoprotein secreted by prostate glandular acinus and is present in the prostatic ductal system, where its blood epithelial barrier will be disrupted following carcinogenesis of the prostate ductal system, resulting in the rising serum content [24]. It was found in this study that the serum T-PSA of treated patients in the combined group was significantly lower than that of the reference group (*P* < 0.001), indicating that the combined therapy could synergistically exert mutual promoting effects and further promote the apoptosis of cancer cells, thereby inhibiting the proliferation of cancer tissues and effectively reducing the content of serum markers, which have been proved in locally advanced prostate cancer [25]. It was also found that compared with the reference group, the mPFS and OS of the combined group were remarkably longer, denoting that the combined therapy worked better in prolonging patients' survival than the single method. The Cox proportional-hazards model is a semiparametric retrospective model, which simultaneously analyzes the effects of numerous factors on survival and is widely used in medical follow-up studies. In addition, Cox proportional-hazards regression models were also adopted to analyze the factors affecting the prognosis of prostate cancer patients, and it was concluded that the affecting factors were Gleason score of 8–10, positive lymphatic metastasis, and single radiotherapy. The Gleason score is an important indicator for evaluating the degree of malignancy of prostate cancer, and ≥8 points indicate a high degree of malignancy and high possibility of bone metastasis or other tissue metastasis; a positive lymph node metastasis indicates that the tumor has undergone malignant transformation; and radiotherapy alone will adversely affect the patients' immune system. Therefore, corresponding clinical preventive measures should be carried out to lower the adverse factors that may affect patient treatment. This study conducted a preliminary efficacy exploration of ADT combined with radiotherapy for the treatment of prostate cancer, but due to the limitation of time and other factors, it still has the following deficiencies: ① no relevant studies on postoperative toxicity in patients were conducted; ② the source of cases was not exclusive, and the number of enrolled cases was small, which might affect the overall efficacy decision of patients; and ③ only the changes in serum T-PSA and VEGF levels in patients were analyzed, but to make the clinical study more scientific, other objective evaluation indexes such as follicle-stimulating hormone (FSH) should be included.

In conclusion, combining ADT with radiotherapy obtains significantly better efficacy than single radiotherapy in treating prostate cancer and is therefore recommended in the clinical treatment of prostate cancer.

## Figures and Tables

**Table 1:** Between-group comparison of baseline data (*n* = 47).

Item	Combined group	Reference group	*X* ^2^/*t*	*P*
Mean age (mean ± SD, years)	63.37 ± 5.47	64.08 ± 5.39	0.634	0.528
BMI (mean ± SD, kg/m^2^)	21.13 ± 4.37	21.17 ± 4.41	0.046	0.964
Prostate volume (mean ± SD, cm^3^)	25.17 ± 3.28	25.13 ± 3.34	0.060	0.952
Disease duration (mean ± SD, month)	7.12 ± 2.14	7.15 ± 2.18	0.069	0.945

TNM pathological stage				
II	25 (53.19%)	28 (59.57%)	0.389	0.533
III	17 (36.17%)	16 (34.04%)	0.047	0.829
IV	5 (10.64%)	3 (6.38%)	0.593	0.441
Accompanied metastasis				
Bone metastasis	25 (53.19％)	22 (46.81％)	0.383	0.536
Lymph node metastasis	16 (34.04％)	12 (25.53％)	0.814	0.367
Viscera metastasis	6 (12.77％)	13 (27.66％)	3.232	0.072
Nationality (*n*(%))			0.712	0.399
Han	43 (91.49%)	45 (95.74%)		
Non-Han	4 (8.51%)	2 (4.26%)		

ECOG score				
0 points	23 (48.94%)	25 (53.19%)	0.170	0.680
1 point	16 (34.04%)	15 (31.91%)	0.048	0.826
2 points	8 (17.02%)	7 (14.89%)	0.079	0.778

Degree of differentiation				
Poor differentiation	14 (29.79%)	17 (36.17%)	0.433	0.510
Moderate differentiation	21 (44.68%)	20 (42.55%)	0.043	0.835
Well differentiation	12 (25.53%)	10 (21.28%)	0.237	0.626

Marital status (*n*(%))				
Single	2 (4.26%)	3 (6.38%)	0.211	0.646
Married	41 (87.23%)	38 (80.85%)	0.714	0.398
Divorced	4 (8.51%)	6 (12.77%)	0.448	0.503
Place of residence (*n*(%))			0.170	0.680
Urban area	24 (51.06%)	22 (46.81%)		
Rural area	23 (48.94%)	25 (53.19%)		

Educational degree [*n*(%)]				
Junior college and above	8 (17.02%)	10 (21.28%)	0.275	0.600
Senior high school	26 (55.32%)	23 (48.94%)	0.384	0.536
Junior high school and below	13 (27.66%)	14 (29.79%)	0.052	0.820

**Table 2:** Between-group comparison of treatment effect (*n*(%), *n* = 47).

Group	CR	PR	SD	PD	ORR (CR + PR)	DCR (CR + PR + SD)
Combined	17 (36.17)	8 (17.02)	19 (40.43)	3 (6.38)	53.19% (25/47)	93.62% (44/47)
Reference	11 (23.40)	4 (8.51)	24 (51.06)	8 (17.02)	31.91% (15/47)	82.98% (39/47)
*X* ^2^					4.352	3.891
*P*					0.037	0.049

**Table 3:** Single-factor retrospective analysis on clinical prognosis.

Factor	*B*	*S*_*b*_	Wald *X*^2^	*P*	OR	95% CI	
						Lower limit	Upper limit
Age	1.364	0.836	3.462	0.463	3.267	0.253	1.732
Pathological stage	2.047	3.276	2.373	0.624	2.165	0.437	1.537
Tumor size	1.564	0.436	2.361	0.253	5.472	0.734	1.345
Treatment method	1.932	2.357	0.836	0.006	4.274	1.323	3.267
Gleason score	2.367	0.924	1.263	0.026	3.274	1.367	2.276
KPS score	1.628	0.637	1.935	0.628	4.274	0.367	2.351
Lymphatic metastasis	0.895	1.924	4.365	0.007	3.574	1.527	2.354

**Table 4:** Multi-factor retrospective analysis on clinical prognosis.

Factor	*b*	*S*_*b*_	Wald *X*^2^	*P*	OR	95% CI	
						Lower limit	Upper limit
Age	1.364	0.836	3.462	0.463	3.267	0.253	1.732
Pathological stage	2.047	3.276	2.373	0.624	2.165	0.437	1.537
Tumor size	1.578	0.436	2.361	0.253	5.472	0.734	1.345
Treatment method	1.932	2.357	0.836	0.018	3.736	1.467	3.327
Gleason score	3.273	1.947	1.037	0.014	3.843	1.426	2.357
KPS score	1.738	0.737	2.374	0.628	4.348	0.367	2.351
Lymphatic metastasis	1.236	1.924	4.247	0.002	2.183	1.683	2.874

## Data Availability

The data used to support the findings of this study are available on reasonable request from the corresponding author.
